# *Lactobacillus plantarum* PS128 Promotes Intestinal Motility, Mucin Production, and Serotonin Signaling in Mice

**DOI:** 10.1007/s12602-021-09814-3

**Published:** 2021-07-29

**Authors:** Chih-Ming Chen, Chien-Chen Wu, Chin-Lin Huang, Min-Yu Chang, Shih-Hsuan Cheng, Ching-Ting Lin, Ying-Chieh Tsai

**Affiliations:** 1 Bened Biomedical Co., Ltd, Taipei, 10448 Taiwan; 2 School of Chinese Medicine, China Medical University, Taichung, 40402 Taiwan; 3 Institute of Biochemistry and Molecular Biology, National Yang Ming Chiao Tung University, Taipei, 11221 Taiwan

**Keywords:** *Lactobacillus plantarum*, PS128; Psychobiotic, Gut–brain axis, Serotonin signaling

## Abstract

*Lactobacillus plantarum* PS128 has been reported as a psychobiotic to improve mental health through the gut–brain axis in experimental animal models. To explore its mechanism of action in the gut, this study aimed to analyze the effects of *L. plantarum* PS128 ingestion on naïve and loperamide (Lop)-induced constipation mice. We found that, in the two mouse models, the weight, number, and water content of feces in the *L. plantarum* PS128 group were higher than those in the vehicle control group. Histological observation revealed that *L. plantarum* PS128 increased the level of colonic mucins including the major mucin MUC2. In addition, the charcoal meal test showed that *L. plantarum* PS128 significantly increased the small intestine transit in naïve mice, but not in the Lop-treated mice. Since intestinal serotonin has been found to modulate motility, we further analyzed the expression of genes related to serotonin signal transduction in the small intestine of naïve mice. The results showed that *L. plantarum* PS128 significantly altered the expression levels of *Tph1*, *Chga*, *Slc6a4*, and *Htr4*, but did not affect the expression levels of *Tph2*, *Htr3a*, and *Maoa*. Furthermore, immunohistochemistry revealed that *L. plantarum* PS128 significantly increased the number of serotonin-containing intestinal cells in mice. Taken together, our results suggest that *L. plantarum* PS128 could promote intestinal motility, mucin production, and serotonin signal transduction, leading to a laxative effect in mice.

## Introduction

Probiotics are defined as live microorganisms which when administered in adequate amounts confer a health benefit on the host [1]. In the market, probiotics have been extensively available as food products (cheese, yogurt, fermented milk, meat, and vegetables) or food supplements (capsules, tablets, and powders). Numerous studies have demonstrated the diverse beneficial effects of probiotics in the maintenance of gastrointestinal (GI) homeostasis [2, 3], regulation of immune responses [4], and attenuation of metabolic dysfunction [5].

The gut–brain axis facilitates bidirectional communication between the GI tract and the brain or between the enteric nervous system (ENS) and central nervous system (CNS), which involves the neural, immune, and endocrine pathways [6]. A special class of probiotics, termed “psychobiotics,” can improve the CNS-related functions and behaviors of the host through the gut–brain axis [7]. Moreover, psychobiotics have been demonstrated to improve the neurodegenerative and neurodevelopmental disorders, including autism spectrum disorder (ASD) and Parkinson’s disease (PD) [8]. Though, psychobiotic effects are considered strain-specific, a given strain might exert several health-promoting effects in many cases [9].

*Lactobacillus plantarum* PS128 is a novel psychobiotic that alleviates depression- and anxiety-like behaviors [10, 11], visceral hypersensitivity [12], and neurobehavioral aspects of movement disorders [13, 14] in experimental animals. Clinically, *L. plantarum* PS128 appears to ameliorate the opposition/defiance behaviors in children with ASD [15], enhance exercise performance in triathletes [16], and improve self-perceived stress and salivary cortisol levels in highly stressed information technology specialists [17]. These studies suggest that *L. plantarum* PS128 affects CNS-related functions through the gut–brain axis. However, the effect of *L. plantarum* PS128 on GI function, including motility and secretion, remains largely unknown.

In this study, we aimed to evaluate the effect of *L. plantarum* PS128 on fecal parameters, intestinal motility, and intestinal secretion in two mouse models, including naïve mice and loperamide (Lop)-induced constipation model mice. Since intestinal serotonin (5-hydroxytryptamine; 5-HT) is a well-known neurotransmitter that regulates GI motility [18], we further studied the effect of *L. plantarum* PS128 on the expression of genes related to serotonin signaling in the intestine.

## Materials and Methods

### Preparation of *Lactobacillus plantarum* PS128 Culture

*Lactobacillus plantarum* (recently re-classified as *Lactiplantibacillus plantarum* [19]) PS128 was prepared using the method described by Liao et al. [14]. In brief, *L. plantarum* PS128 was cultured at 37 °C in the de Man, Rogosa and Sharpe broth (Difco Corp., MD, USA) for 18 h. The bacterial culture was harvested by centrifugation at 6000 × *g* for 10 min. Before oral administration, the bacterial pellet was resuspended in sterile phosphate-buffered saline (PBS) to attain a final concentration of approximately 10^10^ colony-forming units (CFUs)/mL.

### Animals

Eight-week-old adult male ICR mice were purchased from the National Laboratory Animal Center, Taipei, Taiwan. All mice were maintained on a 12-h light/dark cycle in a humidity-controlled (55–65%) and temperature-controlled (22 ± 2 °C) environment with standardized laboratory chow and tap water ad libitum at the National Yang Ming Chiao Tung University Laboratory Animal Center. The use of animals and the procedures for animal handling and treatments were approved by the Institutional Animal Use and Care Committee (IACUC 1,060,606) at the National Yang Ming Chiao Tung University in Taiwan.

### Experimental Design

**Fig. 1:**
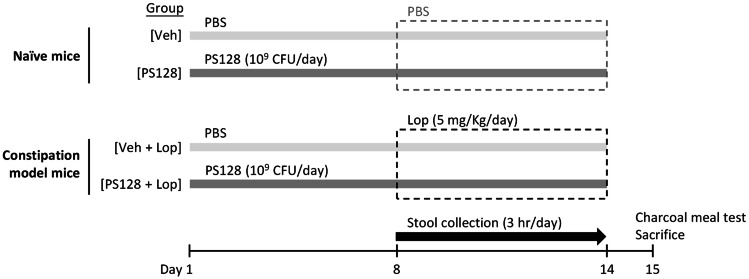
The experimental design. Laxative effects of *L. plantarum* PS128 were evaluated in naïve (upper panel) and constipation model mice (lower panel). Male ICR mice (8-week-old) were orally administered 0.2 mL of PBS, as the vehicle control (Veh) group, or 10^9^ CFU of *L. plantarum* PS128 per day for 14 consecutive days. To induce constipation, mice were orally administered loperamide hydrochloride (Lop. HCl; 5 mg/kg body weight) 1 h after the administration of PBS or PS128 on experimental days 8 to 14. Thirty minutes after the administration of PBS or Lop. HCl, the number and weight of stool from each mouse were measured for 3 h. On experimental day 15, mice were subjected to the charcoal meal test and sacrificed (*n* = 10 per group)

As shown in Fig. 1, mice were divided into four groups (*n* = 10 per group). The PS128 group received daily oral gavage of PS128 of 10^9^ CFU for 14 consecutive days while vehicle control (Veh) group received PBS (0.2 mL per day). The PS128 + Lop group received daily oral gavage of PS128 of 10^9^ CFU for 14 consecutive days, and orally administered loperamide hydrochloride (5 mg/kg body weight; Sigma-Aldrich, St. Louis, MO, USA) on day 8 to 14 of the experiment [20]. In addition, the Veh + Lop group received daily oral gavage of 0.2 mL PBS for 14 consecutive days and the same treatment of Lop. To analyze fecal parameters, the wet weight and number of feces were measured for 3 h per day on day 8 to 14 of the experiment. Subsequently, the collected feces were dried at 70 °C for 18 h and weighed to determine the percentage of water content, which was calculated using the following formula: Fecal water content (%) = [(wet mass − dry mass)/wet mass] × 100. On day 15 of the experiment, the small intestinal transit rate of mice was determined by a charcoal meal test, and the mice were sacrificed for subsequent analysis.

### Charcoal Meal Test

To measure the small intestinal transit rate in mice, a charcoal meal test was performed as previously described with minor modifications [21]. After fasting for 16 h with water ad libitum, mice were orally administered 0.2 mL of PBS or *L. plantarum* PS128 suspensions. After 1 h, mice were administered PBS or Lop. HCl by oral gavage followed by administration of charcoal meal (5% charcoal and 10% gum arabica) after 30 min. Fifteen minutes later, mice were sacrificed by cervical dislocation and the small intestine was removed from the stomach to the caecum to measure the distance traveled by the charcoal meal and total length of the intestine. The small intestine transit rate was calculated using the following formula: small intestine transit rate (%) = (distance traveled by the charcoal meal/total length of the intestine) × 100.

### qRT-PCR Analysis

The total RNA in the ileum tissue was extracted, converted to cDNA, and subjected to quantitative reverse-transcription-polymerase chain reaction (qRT-PCR) analysis as previously described [22]. In brief, cDNA samples from each group were subjected to triplicate real-time PCR experiments with specific primers (Table 1) and KAPA SYBR FAST ABI PRISM Kit (KAPA Biosystems, Woburn, MA, USA) using the StepOnePlus Real-Time PCR System (Applied Biosystems, Foster City, CA, USA). The targets included genes encoding tryptophan hydroxylase 1 and 2 (*Tph1* and *Tph2*), chromogranin A (*Chga*), the serotonin transporter solute carrier family 6 member 4 (*Slc6a4*), 5-hydroxytryptamine receptor 3A and 5-hydroxytryptamine receptor 4 (*Htr3a* and *Htr4*), and monoamine oxidase A (*Maoa*). The target threshold cycle (Ct) was subtracted from the Ct for glyceraldehyde-3-phosphate dehydrogenase (*Gapdh*) to calculate ΔCt, and a relative quantification analysis was performed using the 2^−ΔΔCT^ method.

**Table 1:** Primers used in the present study

Gene	Primer sequence (5′–3′)	Reference
*Tph1*	F: TTTCGAGTCTTTCACTGCACT	[57]
	R: CTAGGAGTTCATGGCAGGT	
*Tph2*	F: GAGTTGCTCCACGCTTTGC	[58]
	R: ACACTCAGTCTACATCCATCCC	
*Chga*	F: CCCACTGCAGCATCCAGTT	[59]
	R: AGTCCGACTGACCATCATCTTTC	
*Slc6a4*	F: TATCCAATGGGTACTCCGCAG	[60]
	R: CCGTTCCCCTTGGTGAATCT	
*Htr3a*	F: TGACCGCCTGTAGCCTTGAC	[61]
	R: TCCCACTCGCCCTGATTTATG	
*Htr4*	F: AGTTCCAACGAGGGTTTCAGG	[62]
	R: CAGCAGGTTGCCCAAGATG	
*Maoa*	F: GGAGAAGCCCAGTATCACAGG	[63]
	R: GAACCAAGACATTAATTTTGTATTCTGAC	
*Gapdh*	F: CAATGTGTCCGTCGTGGATCT	[64]
	R: GTCCTCAGTGTAGCCCAAGATG	

*F* forward primer, *R* reverse primer

### Histological Analysis

The paraffin-embedded distal colon and ileum tissue blocks were sectioned into 5-μm-thick slices and mounted on poly(lysine)-coated slides. After deparaffinization and rehydration, tissue sections were further subjected to alcian blue staining, and the expression levels of MUC2 and 5-HT were determined by immunohistochemical analysis. For alcian blue staining analysis, distal colon sections were rinsed with 3% acetic acid for 3 min and then incubated with 1% alcian blue solution (pH 2.5) for 15 min. After running tap water for 5 min, the sections were subjected to neutral red staining for 1 min. Slides were mounted and visualized under a microscope.

For immunohistochemical analysis, the expression levels of MUC2 and 5-HT in the distal colon and ileum were detected. The paraffin sections were deparaffinized, blocked with 3% hydrogen peroxide for 10 min, and subjected to antigen retrieval with microwaves in a 0.01 M citrate buffer for 15 min. The slides were then washed twice with PBS and incubated with MUC2 (1:100; ab76774, Abcam, UK) or 5-HT (1:1000; #20,080, Acris, Herford, Germany) antibodies. This was followed by incubation with the polymer conjugated peroxidase for 30 min using a polymer detection system (Zymed Laboratories, San Francisco, CA, USA). Finally, the color was developed using 3, 3-diaminobenzidine (Sigma, St. Louis, MO, USA). The slides were counterstained with Gill’s hematoxylin (Sigma-Aldrich, St. Louis, MO, USA), then dehydrated, and mounted prior to microscopic reading.

Images were observed and photographed using a microscope equipped with a digital image system. Quantitative analyses were performed using ImageJ software, for alcian blue- and MUC2- positive area, or by counting 5-HT-positive cells under five different fields to calculate the mean ± standard error of means (SEM) per filter.

### Statistical Analysis

Data were analyzed using GraphPad Prism (GraphPad Prism, version 7, La Jolla, CA, USA) and represented as means ± SEM. The changes in the fecal parameters over time between two groups (vehicle control and probiotic groups) were analyzed using two-way analysis of variance (ANOVA), mixed design. For multiple comparisons, one-way ANOVA with Tukey’s post hoc test was used. Statistical significance was set at *P < 0.05.

## Results

### Increased Fecal Output in Mice Treated with *L. plantarum* PS128

**Fig. 2:**
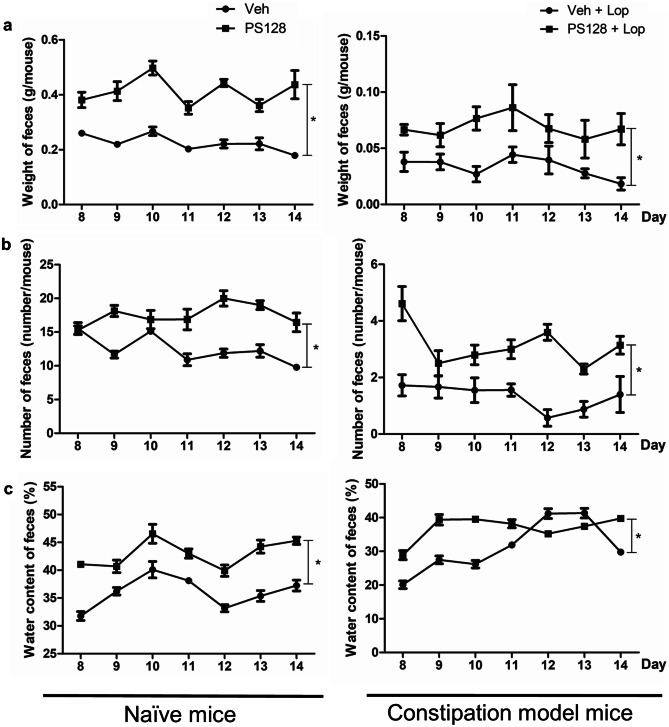
Effects of *L. plantarum* PS128 on fecal parameters in naïve and Lop-induced constipation model mice. Mice were orally administered PBS or 10^9^ CFU of *L. plantarum* PS128 for 14 consecutive days. On experimental days 8 to 14, the wet weight (**a**), number (**b**), and water content (**c**) of feces were measured for 3 h. Data were expressed as mean ± SEM. Differences between groups were analyzed using two-way ANOVA, mixed design. *P < 0.05 compared with the indicated groups (*n* = 10 per group)

The effects of *L. plantarum* PS128 on fecal parameters were analyzed in naïve and Lop-treated mice. Compared with the vehicle control (Veh) group, PS128 ingestion resulted in increased fecal weight (Fig. 2a), number (Fig. 2b), and water content (Fig. 2c) in both mouse models, suggesting a laxative effect of *L. plantarum* PS128.

### Histology of the Distal Colon

**Fig. 3:**
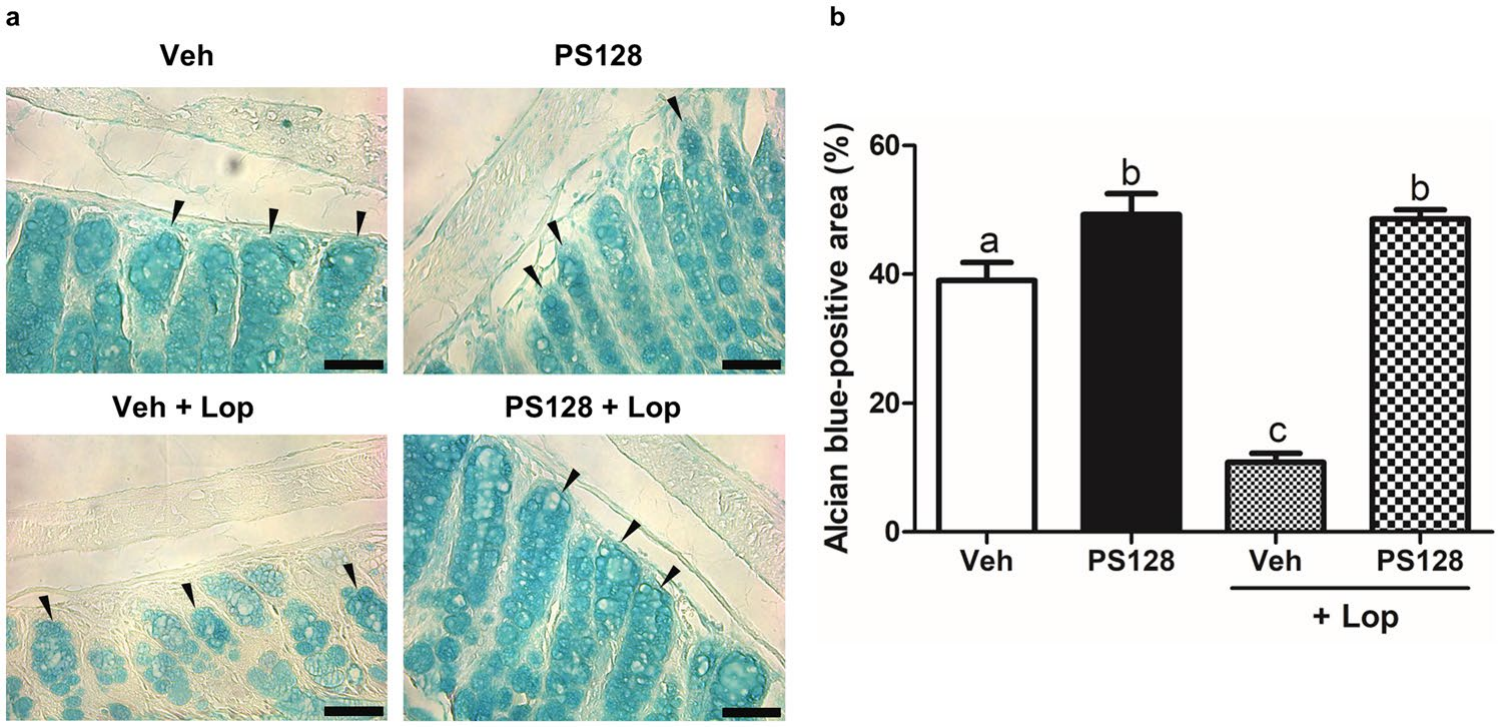
Oral administration of *L. plantarum* PS128 increased the amount of colonic mucus in mice. **a** Representative images of Alcian blue stained areas of distal colonic sections (some are indicated by black arrows). Bars, 50 μm. **b** Quantification using the ImageJ software for each group (*n* = 6 per group). Data were expressed as mean ± SEM and analyzed by one-way ANOVA with Tukey’s post hoc test, and different superscript letters (a, b, c) differed significantly at *P* < 0.05

**Fig. 4:**
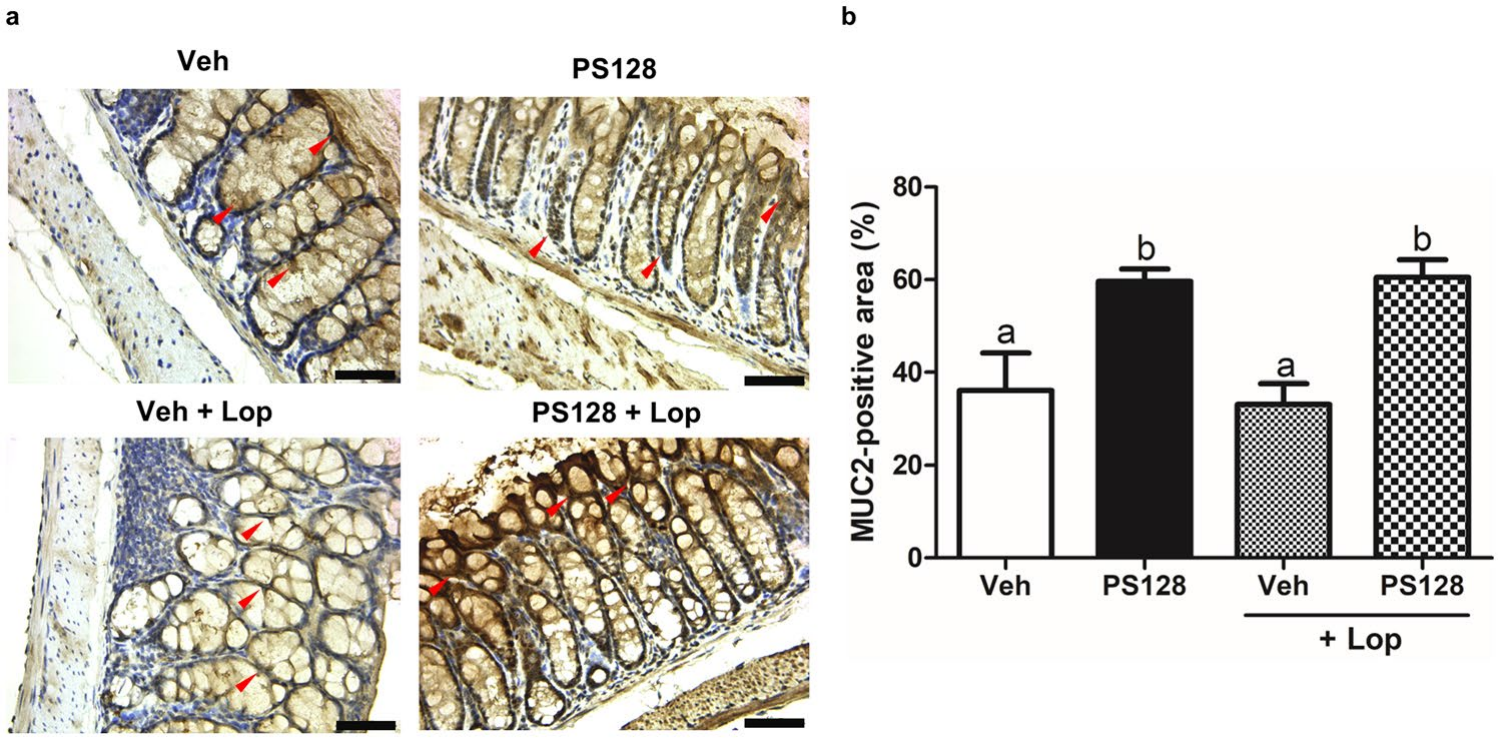
Oral administration of *L. plantarum* PS128 increased the amount of colonic MUC2 in mice. **a** The distal colons were analyzed for the expression of MUC2 by immunohistochemistry (some are indicated by red arrows). Bars, 50 μm. **b** Quantification using the ImageJ software for each group (*n* = 6 per group). Data were expressed as mean ± SEM and analyzed by one-way ANOVA with Tukey’s post hoc test, and different superscript letters (a, b) differed significantly at *P* < 0.05

Alcian blue staining was performed to analyze mucin production in the distal colon (Fig. 3a). As shown in Fig. 3b, image quantification of the stained areas showed that, compared with the Veh group, PS128 significantly increased the amount of colonic mucin in naïve mice. In addition, treatment with Lop significantly reduced the amount of colonic mucin, which could be reversed by the ingestion of PS128. Furthermore, immunohistochemical detection and image quantification of MUC2, the major intestinal mucin, showed that PS128 significantly increased the level of MUC2 in both naïve and Lop-treated mice (Fig. 4). However, treatment with Lop did not affect the level of MUC2 in the distal colon.

### *L. plantarum* PS128 Increases the Small Intestine Transit Time in Naïve Mice

**Fig. 5:**
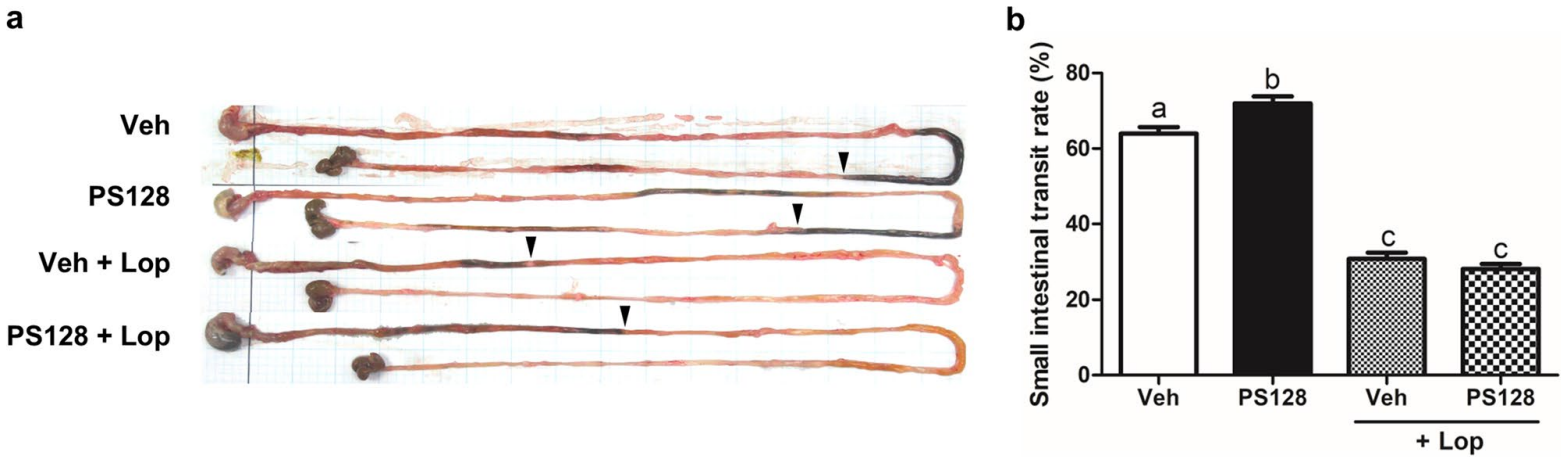
Effect of *L. plantarum* PS128 on the small intestinal transit rate assessed by the charcoal meal test. **a** An actual image of the charcoal meal transit site in the small intestine. The stomach (left side), small intestine, and cecum were excised from mice, and their morphology was observed using a digital camera. The black arrows indicate the position of the charcoal meal. **b** Small intestinal transit rate for each group treated with charcoal meal (*n* = 6 per group). Data were expressed as mean ± SEM and analyzed by one-way ANOVA with Tukey’s post hoc test, and different superscript letters (a, b, c) differed significantly at *P* < 0.05

To investigate whether *L. plantarum* PS128 affects intestinal motility, a charcoal meal test was performed. As shown in Fig. 5a, a representative photograph shows the total length of the small intestine and the distance traveled by the charcoal meal in the intestine. Compared with the Veh group, PS128 significantly increased the small intestinal transit rate in naïve mice (Fig. 5b). In addition, treatment with Lop significantly reduced the small intestinal transit rate. However, this reduction could not be ameliorated by PS128. Since PS128 only increased the intestinal motility in naïve mice, but not in Lop-treated mice, we focused on two groups of naïve mice to further analyze how PS128 affects the intestinal motility.

### *L. plantarum* PS128 Modulates the Serotonin Signal Transduction in the Intestine

**Fig. 6:**
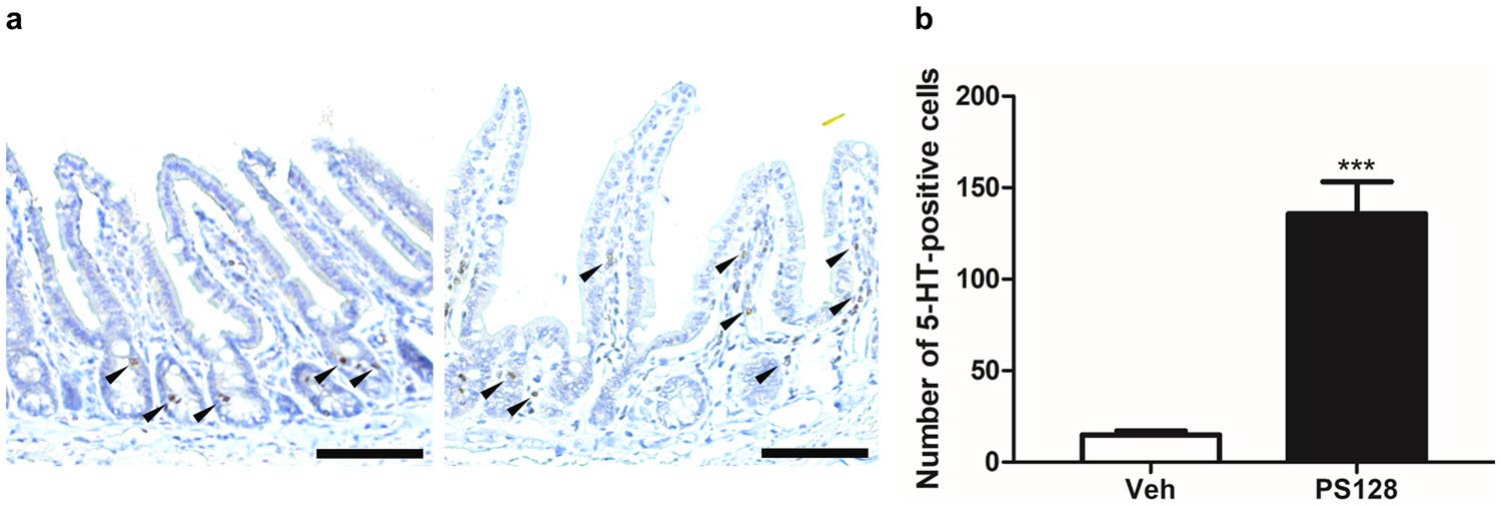
Oral administration of *L. plantarum* PS128 increased the number of 5-HT-positive cells in the ileum of naïve mice. **a** Representative images showing 5-HT-positive cells in the ileum tissues from mice. The 5-HT-positive cells were indicated by black arrows. Bars, 5 μm. **b** Numbers of 5-HT-positive cells per area in the ileum tissues of mice (*n* = 4 per group). Data were expressed as mean ± SEM and analyzed using unpaired *t* test. ***P < 0.001 compared with the vehicle control groups

To further investigate the mechanism of action of *L. plantarum* PS128 on intestinal motility, qRT-PCR was performed to analyze the expression of genes related to serotonin signal transduction (Table 1). As shown in Table 2, compared with the Veh group, PS128 ingestion significantly increased *Tph1* expression and decreased the expression levels of *Chga*, *Slc6a4*, and *Htr4*. In addition, no other significant differences in the expression levels of *Tph2*, *Htr3a*, and *Maoa* were observed. Furthermore, we examined 5-HT expression in the ileum sections by immunohistochemical staining (Fig. 6a). Compared with the Veh group, the number of 5-HT-positive cells was significantly increased in the PS128 group (Fig. 6b).

**Table 2:** Gene expression of 5-HT related genes in the ileum of naïve ICR mice

Gene	Vehicle	PS128
*Tph1*	1.0 ± 0.40	3.2 ± 1.18***
*Tph2*	1.0 ± 0.67	0.8 ± 0.87
*Chga*	1.0 ± 0.53	0.7 ± 0.31*
*Slc6a4*	1.0 ± 0.46	0.5 ± 0.15***
*Htr3a*	1.0 ± 0.61	1.0 ± 0.70
*Htr4*	1.0 ± 0.93	0.2 ± 0.17***
*Maoa*	1.0 ± 0.44	1.2 ± 0.29

Gene expression levels of *Tph1*, *Tph2*, *Chga*, *Slc6a4*, *Htr3a*, *Htr4*, and *Maoa* were quantified by real-time PCR relative to the expression of *Gapdh*. Values were expressed as mean ± SEM and analyzed using an unpaired *t* test

^*^P < 0.05; **P < 0.01; ***P < 0.001 versus the vehicle control group

## Discussion

Emerging evidence has demonstrated that intestinal homeostasis affects CNS-related functions through the gut–brain axis in a bidirectional manner [23]. Patients with psychiatric and neurological disorders, including ASD [24], major depressive disorder [25], PD [26], and Alzheimer’s disease [27], are commonly reported to have GI symptoms and imbalanced gut microbiota. Moreover, psychological stress leads to several GI symptoms and plays a crucial role in the development of irritable bowel syndrome (IBS) [28]. In contrast, dysregulated GI mucus secretion and increased intestinal permeability may lead to systemic inflammation and impairment of the blood–brain barrier (BBB), thus negatively influencing CNS-related functions [29]. Therefore, maintaining intestinal homeostasis and improving gut health may help to alleviate CNS disorders. Probiotic food supplementation can reduce stress-induced GI symptoms in volunteers [30], decrease depression scores, alter brain activity in patients with IBS [31], and improve symptoms associated with ASD and PD [8]. These probiotics that affect CNS-related functions and behaviors are classified as psychobiotics, including *L. plantarum* PS128, *Bifidobacterium longum* 1714 [32], *B. longum* NCC3001 [31], *Lactobacillus rhamnosus* JB-1 [33], *Lactobacillus helveticus* R0052, and *B. longum* R0175 [30]. Although these psychobiotics have been suggested to regulate CNS-related functions through the gut-brain axis, their regulation in the intestine remains largely unknown.

In this study, we used two mouse models, naïve and Lop-induced constipation mice, to investigate the effect of *L. plantarum* PS128 on intestinal homeostasis. Daily administration of *L. plantarum* PS128 appeared to increase the fecal output, colonic mucin production, and intestinal motility in naïve mice (Figs. 2, 3, 4, and 5), suggesting that *L. plantarum* PS128 can be used as a laxative food supplement. A recent clinical study has also shown that daily administration of *L. plantarum* PS128 for eight weeks significantly improved self-perceived stress and GI symptoms in highly stressed information technology specialists [17], which supported the results of this study. However, in mice with Lop-induced constipation, the laxative effect of *L. plantarum* PS128 was relatively faint (Fig. 2). Lop has been widely used in animal experiments to reduce intestinal motility and colonic water secretion, thus prolonging the evacuation time of feces [34]. Lop treatment in the Sprague–Dawley rats also resulted in a thinner mucus layer at the fecal surface [35]. In this study, we found that treatment with Lop significantly reduced the level of colonic mucins stained with alcian blue (Fig. 3), which is a central feature of this constipation model [20, 36]. Several studies have also shown that treatment with Lop reduced the expression of colonic MUC2 at mRNA [37, 38] and protein levels [39]. However, the effect of Lop treatment on the expression of MUC2 cannot be observed in this study (Fig. 4). Possible reasons include that, first, alcian blue and MUC2 antibodies detect different targets, which may lead to different results. The staining of alcian blue is restricted mainly to acidic carbohydrates but not the protein core of mucin [40]. Second, although MUC2 is the major colonic mucin, the expression of other mucins, such as MUC3 and MUC6 [41], may affect the results. Third, there are some differences in the response of ICR mice derived from different sources to Lop treatment [36]. It is still unclear how Lop treatment may affect the expression and glycosylation of different colonic mucins.

As shown in Fig. 5, Lop significantly reduced the small intestinal motility; however, this reduction could not be reversed by the administration of *L. plantarum* PS128. These findings indicate that *L. plantarum* PS128 may be ineffective in patients with serious or chronic constipation. Chronic constipation is known to have many possible causes, including blockage of the colon or rectum, dysfunction of the ENS, difficulty in moving the pelvic muscle involved in elimination, and imbalance of hormones in the body. We assume that *L. plantarum* PS128 is only effective for specific subtypes of constipation, and this assumption requires further investigation. On the other hand, pharmacological therapy for constipation includes the use of bulking agents, osmotic agents, stool softeners, stimulant laxatives, lubricants, etc. [42], while non-pharmacological therapy includes increased intake of dietary fibers and water, increased physical activity, and supplementation of probiotics [43, 44]. Fermented milk containing *Lactobacillus casei* Shirota increases bowel movement frequency and stool consistency in patients with constipation [45, 46]. In addition, daily consumption of *L. helveticus* for one week alleviates constipation-related symptoms and reduces both fecal pH and intestinal transit time in patients with constipation-predominant IBS [47]. The efficacy of probiotic products is suggested to be both strain-specific and disease-specific [48], which should be proven by further evidence-based research. Clinically, whether *L. plantarum* PS128 is effective in constipation and PD- and ASD-complicated gut dysfunction remains to be studied.

GI epithelial cells sense luminal signals from the ingested food and microbiota, and then translate and deliver signals to exert local and systematic effects. One of the important signals is 5-HT, which originates from the enterochromaffin (EC) cells of the GI tract, which act as the major source of peripheral 5-HT by secreting 95% of total 5-HT in the body [49]. Although peripheral 5-HT does not cross the BBB [50], gut-derived 5-HT can be carried and released by circulating platelets, remain free in the serum, and interact with the CNS through the ENS, thus affecting various biological phenomena, including gut motility and secretion, bowel inflammation, bone development, and platelet aggregation [51]. In this study, we found that *L. plantarum* PS128 modulated the expression levels of genes related to serotonin signaling in the intestine (Table 2). TPH1 is a key enzyme for 5-HT biosynthesis and Chromogranin A (ChgA) is colocalized with 5-HT in EC cell storage granules [52, 53]. The upregulation of *Tph1* and downregulation of *ChgA* suggested that there is an increase in the biosynthesis and storage of 5-HT in EC cells. Moreover, immunohistochemical analysis showed that *L. plantarum* PS128 increased the number of 5-HT-containing cells in the ileum (Fig. 6). These results support the previous finding that *L. plantarum* PS128 ingestion can increase 5-HT levels in the ileum, colon, and serum of rats [13]. Besides, a previous study has shown that heat-killed *L. casei* 327 promotes colonic 5-HT biosynthesis and GI motility in mice [54], which is similar to the effects exerted by *L. plantarum* PS128. However, heat-killed *L. plantarum* PS128 is ineffective in mice [11]. Thus, we suggest that *L. plantarum* PS128 regulates intestinal 5-HT biosynthesis and motility through mechanisms different from those of heat-killed *L. casei* 327, which may include bacterial secretory molecules, heat-labile constituents, and specific metabolic activities. In addition, *L. plantarum* PS128 downregulates the expression of *Slc6a4* and *Htr4* in the ileum (Table 2), which suggested that 5-HT reuptake would be reduced and may compromise 5-HT_4_ receptor signaling. Correlated expressions of *Slc6a4* and *Htr4* in the mouse colon have been previously reported, which is relevant in the pathogenesis of visceral hypersensitivity by influencing local 5-HT abundance/availability [55]. Moreover, a previous study also reported that *L. plantarum* PS128 alleviated visceral hypersensitivity induced by subcutaneous injection of 5-hydroxytryptophan (5-HTP), a precursor of 5-HT, in rats [56]. Therefore, modulation of the peripheral serotonin signal transduction may be a crucial action mechanism of *L. plantarum* PS128, and this requires further investigation.

This study had several limitations. First, the psychobiotic effects of *L. plantarum* PS128 on the two mouse models were not evaluated; thus, PS128 regulation in the intestine cannot be directly correlated to the host behavior and previous findings in the CNS. Second, although the qRT-PCR results showed that PS128 modulated the expression of 5-HT-related genes in the intestine, the expression level of the protein was not analyzed. Moreover, the mechanism by which PS128 modulates the intestinal serotonin signaling remains largely uncharacterized. Third, the specific role of gut microbiota in this process remains unknown. Future studies of *L. plantarum* PS128 action mechanisms should focus on both the gut and the brain, and experiments using vagotomy or 5-HT agonists/antagonists should be conducted to obtain a more comprehensive understanding of these mechanisms.

## Conclusions

In this study, we found that daily administration of the psychobiotic strain *L. plantarum* PS128 could increase the fecal output, colonic mucin production, and small intestinal motility in mice. Moreover, *L. plantarum* PS128 appeared to promote serotonin signal transduction in the intestine, which might indirectly affect the CNS-related functions and host behaviors through the gut–brain axis.

## Data Availability

The datasets generated during and/or analysed during the current study are available from the corresponding author on reasonable request.
